# Morphological Analysis of White Cement Clinker Minerals: Discussion on the Crystallization-Related Defects

**DOI:** 10.1155/2016/1259094

**Published:** 2016-05-25

**Authors:** Mohamed Benmohamed, Rabah Alouani, Amel Jmayai, Abdesslem Ben Haj Amara, Hafsia Ben Rhaiem

**Affiliations:** ^1^UR05/13-01, Physique des Matériaux Lamellaires et Nanomatériaux Hybrides (PMLNMH), Faculté des Sciences de Bizerte, 7021 Zarzouna, Tunisia; ^2^Département de Géologie, Faculté des Sciences de Bizerte, Zarzouna, 7021 Bizerte, Tunisia

## Abstract

The paper deals with a formation of artificial rock (clinker). Temperature plays the capital role in the manufacturing process. So, it is useful to analyze a poor clinker to identify the different phases and defects associated with their crystallization. X-ray fluorescence spectroscopy was used to determine the clinker's chemical composition. The amounts of the mineralogical phases are measured by quantitative XRD analysis (Rietveld). Scanning electron microscopy (SEM) was used to characterize the main phases of white Portland cement clinker and the defects associated with the formation of clinker mineral elements. The results of a study which focused on the identification of white clinker minerals and defects detected in these noncomplying clinkers such as fluctuation of the amount of the main phases (alite (C3S) and belite (C2S)), excess of the free lime, occurrence of C3S polymorphs, and occurrence of moderately-crystallized structures are presented in this paper.

## 1. Introduction

Portland cement is a mixture of clinker (artificial rock from cooking a vintage mixture of limestone and clay) and ground gypsum (controller plug). The morphology and composition of the phases in a clinker can vary significantly depending on the manufacturing process and raw materials used [1]. In the clinker, the following predominate chemicals elements are Ca, Si, Al, Fe, Mg, Na, and K. These elements are expressed as a percentage of oxides. These elements are expressed as a percentage of oxides and the Bogue [2, 3] notation is used to refer to them: CaO = C, Al_2_O_3_ = A, SiO_2_ = S, Fe_2_O_3_ = F, and MgO = M. Clinker is a multiphase mixture and, so far, more than 30 constituent phases have been identified [4]. Despite the wide variety of clinker phases, only four of them are, in practice, of real importance: silicates including alite (C3S = Ca_3_SiO_5_ = 3CaO-SiO_2_) give the hydrated cement short-term resistance; belite (C2S = Ca_2_SiO_4_ = 2CaO-SiO_2_) which confers long-term resistance to the finished product [5]; aluminates consisting of tricalcium aluminate (C3A = Ca_3_Al_2_O_6_ = 3CaO-Al_2_O_3_) and aluminoferrites (C4AF = Ca_4_Al_2_ Fe_2_O_10_ = 4CaO-Fe_2_O_3_-Al_2_O_3_) [6]. Consider

(1) C3S=Ca3SiO5,C2S=Ca2SiO4,C3A=Ca3Al2O6,C4AF=Ca4Al2Fe2O10.

Depending on the temperature and impurities, C3S has seven polymorphic phases: three triclinic, three monoclinic, and a rhombohedral one [6, 7]. C2S has five polymorphic forms denoted as *γ*, *β*, *α*′L, *α*′H, and *α* in the temperature range between room temperature and 1500°C [7]. The C2S phase usually found in clinker is the *β* monoclinic one [8]. For aluminates, the C3A is frequently found with cubic and orthorhombic forms [9]. The ferrite crystallizes into an orthorhombic form [10]. It is difficult to distinguish between the different interstitial aluminate phases (C3A and C4AF) [11]. Nevertheless, the clinkers usually contain some amount of free lime (up to 1-2%) and free calcium sulphate [12].

In white cement, the whiteness index is a very important parameter to control. However, white cement contains a very low amount of iron since it decreases the whiteness, which implies insignificant content of C4AF. Therefore, white clinker contains three major constituents: C3S, C2S, and C3A [11].

The minerals formed at low temperature (ca 1200–1300°C) are ill crystallized and contain large amounts of admixtures [13]. Their size is usually very small, less than 5 *μ*m, since the clinker crystals are primarily formed by a solid state reaction [14].

Most of the SEM studies to date have dealt with the general characterization of clinker phases, including their compositional variations [15]. Among the articles describing use of the SEM in analysis of clinker microstructure is the work of [16, 17] in which the various clinker phases are identified and described in order to interpret the manufacturing process. Other publications describe the use of SEM in the clinkers minerals analysis [15, 18–27].

With the development of research, we can now ensure that X-ray powder diffraction, combined with the Rietveld [28] method, is the most recent and most accurate way of quantifying the mineralogical composition of Portland clinker [4, 8, 11, 29–37].

However, in some cases, the industry does not comply with the regulations in force when manufacturing clinker. For economic and environmental reasons, the clinker should be recycled back into the original raw material. So any defect in the clinker will be added to the energy cost. This paper uses several analytical techniques (SEM, XRD, and XRF) for the analysis of some poor cement clinkers, aiming to identify different phases and defects within the clinkers.

## 2. Experimental

### 2.1. Materials

Clinker samples (SO1–SO6) and raw materials samples were taken from SOTACIB (Tunisian-Andalusian White Cement Company). They were subjected to the following analysis: a chemical analysis by X-ray fluorescence (ARL type XP 9800) to determine their compositions expressed as oxides in wt.%. Scanning electron microscopy (JEOL JSM-5400) was carried out to identify the mineralogical phases of the clinkers and the associated defects. Finally, a quantitative phase analysis was performed using X-ray powder diffraction (Bruker D8 ADVANCE Diffractometer) and the Rietveld method.

### 2.2. Sample Preparation

#### 2.2.1. X-Ray Fluorescence (XRF)

The same ground samples were used to determine the geochemical compositions. We started by measuring the loss on ignition (LOI) for each sample. Then, in a platinum crucible, 1 g of decarbonated material was added to 6 g lithium tetraborate. The crucible was placed in a furnace at 1100°C for 20 min while melts were stirred every 5 minutes. A mould was placed in the furnace for 5 min and the melts in the crucible were then poured into it. The resulting pearls were analyzed by X-ray fluorescence (XRF).

#### 2.2.2. X-Ray Diffraction (XRD)

All samples were finely ground (down to ~10 *μ*m) for the powder diffraction measurements. XRD data were collected at room temperature using Cu-K*α* radiation (*λ* = 1.5406 Å) operated in the reflection geometry (*θ*/2*θ*). Data were recorded from 10° to 60° (2*θ*) with a step-size of 0.02. The X-ray tube was operated at 40 kV and 40 mA.

#### 2.2.3. Scanning Electron Microscopy

The clinker compounds studied were examined by scanning electron microscopy (SEM). The same samples as those for XRD were analyzed by SEM, but in the latter case they were not ground. Instead, grains of the order of several millimeters in diameter were used for SEM analysis.

## 3. Results and Discussion

The raw material is represented by the chalky limestone of Jebel Feriana (Tunisia) of Abiod from Campanian-Maastrichtian age, silica sands of Beglia (Tunisia) from Miocene age and kaolin of Turkey (imported).

*Limestone*. This is a white limestone from the Campanian-Maastrichtian age, low hardness, which has a high content of CaCO_3_ (>95%) and minimal amounts of colorants metal oxides, mainly Fe_2_O_3_, Ti_2_O_3_. This creates a fairly high degree of whiteness (*β* ≈ 85.34) (Table 1). 

*Purity*. Compared to the calcite containing 56% of CaO, the limestone of Jebel Feriana has a mean content of 98.16% CaCO_3_. 

*Kaolin*. The kaolin used by SOTACIB is imported from Turkey. It meets the quality requirements expressed in a specified set of specifications. Imported kaolin is rich in silica; it contains a mean of 58.52%. While it is poor in alumina, it contains a 26.07% mean content of Al_2_O_3_. The main metal oxides are present in minimal concentrations of 0.85% for Fe_2_O_3_ and 0.79% for titanium (TiO_2_) (Table 1). 

*Sand*. The Sands of Beglia are extrasilica sand (SiO_2_ > 90%). Coloring oxides are presented with very low concentrations as shown in Table 1.

Generally, in the white cement, limestone acts to a weight of about 4 times higher than that of sand and kaolin (about 80% limestone, 10% kaolin, and 10% sand). But it is unstable because of the variation of the geochemical composition of raw materials. In light of crude's control parameters, namely, LSF (between 96 and 97%) and MS (in the order of 5.3%); and alumina (2.6 to 2.7%), the flow rate of material was modified to achieve a geochemical composition of regular crude in time and complying with the standards.

**Figure 1:**
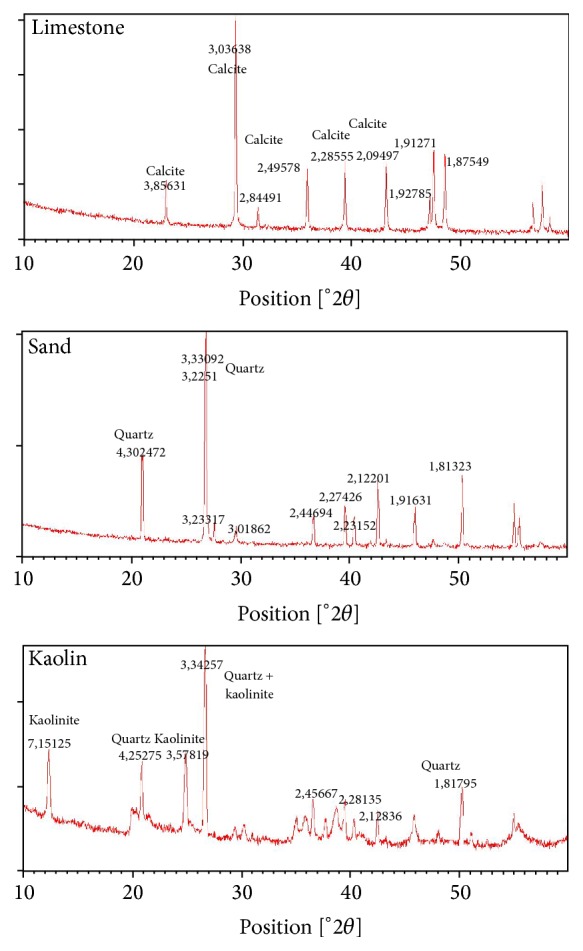
XRD of the samples of raw materials.

The XRD data showed the main mineralogical components of different raw material used for white cement clinker production (Figure 1).

The values for the weight percentage of oxides are listed in Table 2. The compositions of clinkers studied were distributed as follows: lime and silica (CaO ≈ 70% and SiO_2_ ≈ 23-24%), alumina (Al_2_O_3_ ≈ 3-4%), and other minor elements (Fe_2_O_3_, K_2_O, SO_3_, TiO_2_, and MgO, all present in small quantities).

**Figure 2:**
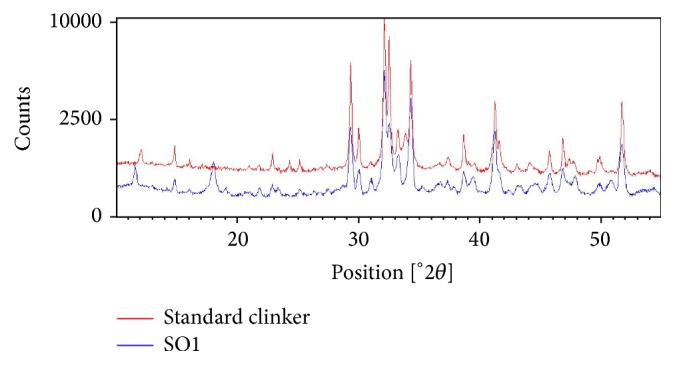
XRD pattern of sample 1 (SO1) clinker (blue) and a good quality standard clinker (red).

**Figure 3:**
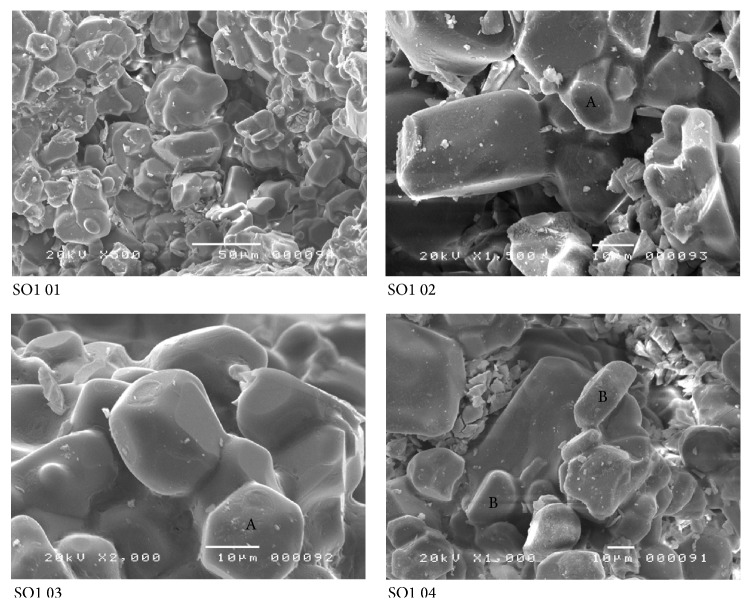
SEM observations of the SO1 sample: (A: alite and B: belite).

The XRD data showed the effect of clinker defects when analyzing the X-ray diffraction peaks. The comparison of the full width at half maximum (FWHM) values for different peaks of the (SO1) sample diffraction pattern and that of a standard clinker shows this effect (Figure 2). In fact, peak broadening was observed in the sample diffraction. Furthermore, the DRX pattern of sample SO1 presents peaks less intense than those of standard clinker, which indicates the incomplete crystallization of the calcium silicate. This is an indication of the poor quality of the clinker as well as the poor formation of the mineralogical phases. The scanning electron micrograph (Figure 3) shows that this clinker presents crystals with hexagonal and circular shapes for alite and belite, respectively [43]. The SEM observations of the samples SO1 (Figure 3) showed likewise, globular shapes with truncations and invisible crystal edges. It is also important to note the presence of voids on either side of the crystal, often employed by the uncombined lime, as well as the irregular and interconnected pores.

The X-ray data was refined by Rietveld analysis using the X'Pert HighScore Plus program (from PANalytical). The Rietveld analysis always gives the sum of the phases present normalized to 100%. Therefore, all the phases present must be entered into the analysis with their known crystal structure.

Crystal structures of alite [38], belite [39], cubic C3A [9], free lime [40], portlandite [41], and periclase [42] taken from the literature were used for refinement and quantification.

XRD profiles were fitted using the pseudo-Voigt function.

The Rietveld refinement is carried out in the following order:

Background (polynomial of 5 coefficients combined with 1/*X* term), zero shift (2*θ*), scale factor, and cell parameter were refined first, followed by the phase profile parameters (*W*, *U*, and *V*).

**Figure 4:**
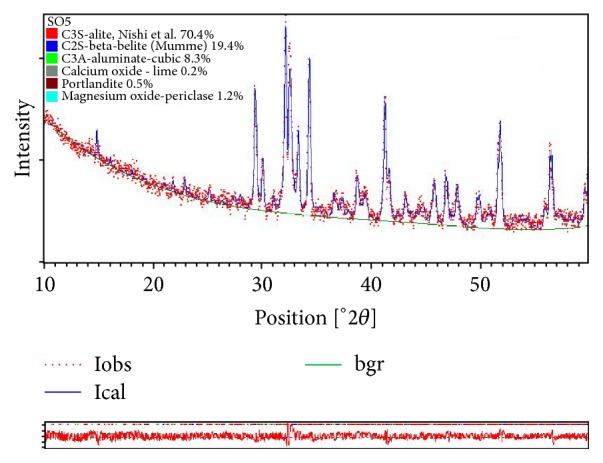
Rietveld refinement of the (SO5) sample.

An example of the Rietveld fitting (for sample SO5) is displayed in Figure 4.

The calculated wt.% of the phases and the Rietveld conventional agreement indexes are listed in Table 3.

The quantification of poor white Portland clinkers by the Rietveld method showed the abundance of two major phases (C3S-monoclinic and C2S-monoclinic (beta)). These two phases showed significant variation from one sample to another. The abundance of lime in sample (SO3) was also noted Table 3. The fluctuations in the contents of C3S and C2S (C3S: 58.5–85.8%; C2S: 2.8–27.6%) can be explained by changes in the kiln temperature (cold kiln). The levels variability of *β*-C2S (belite-monoclinic) also indicates the instability of the kiln temperature and noncontrolled cooling. The occurrence of high percentage of portlandite (SO3 and SO4; Table 3) suggested noncontrolled cooling.

### 3.1. Characterization of Clinker Phases by SEM

SEM analysis was carried out in order to recognize different phases (crystalline and amorphous phases) within the clinker. This analysis compared the cooking state between clinkers. SEM also helped to interpret the burning condition.

#### 3.1.1. Observations

For some samples, the crystalline phases were identified to be mainly alite and belite:

- The crystals had well-defined geometric shapes. The presence of blunt, cracked, ovoid, and anhedral shapes was also observed [20]. In addition, large flattened crystals were also detected.
- Belite was also present in many forms. This is often associated with large, flat alite crystals.
- Lime, with a whish appearance and flower-like structure, existed near the newly formed crystals and in the voids.
- In addition, the presence of anhedral particles can be due to silica gel which formed isolated crystals.

These findings suggest that the investigated clinker, fired at unstable temperature kiln (produced under various conditions), presents different morphologies and sizes. These results are reported of the problems with cooking and excess lime and silica in the raw material.

#### 3.1.2. Minerals

**Figure 5:**
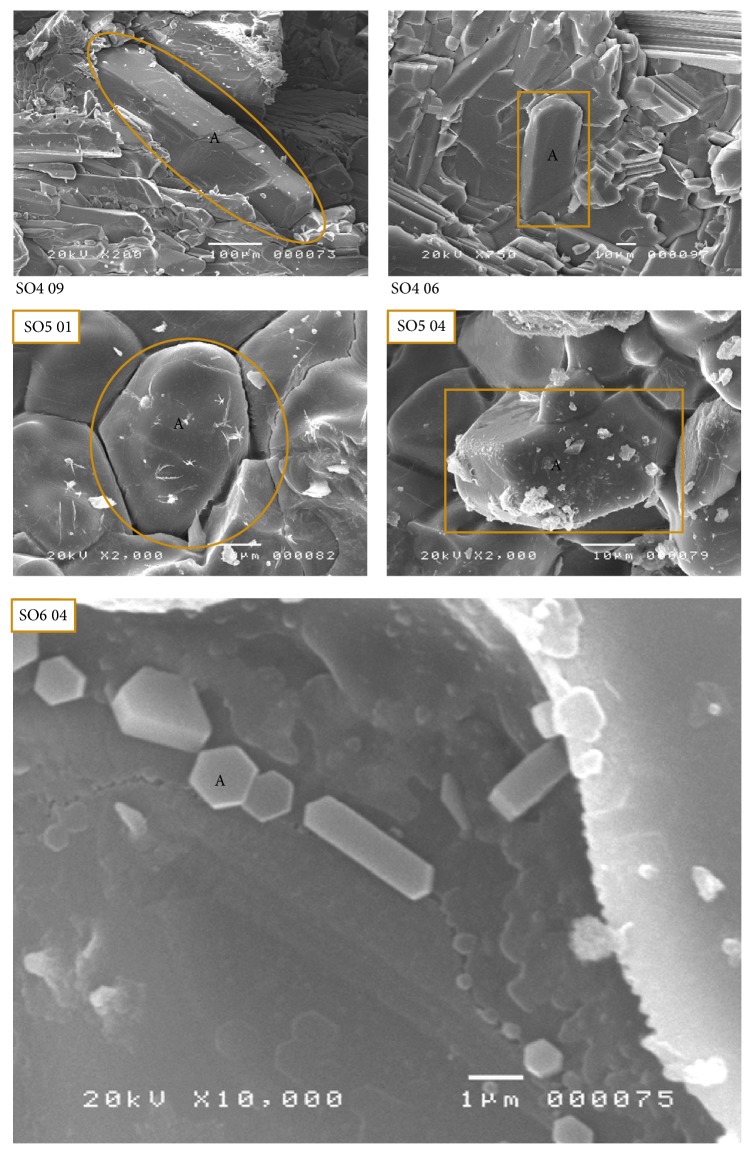
Scanning electron micrograph shows different morphologies of alite mineral (C3S) found in white clinker (A: alite).

**Figure 6:**
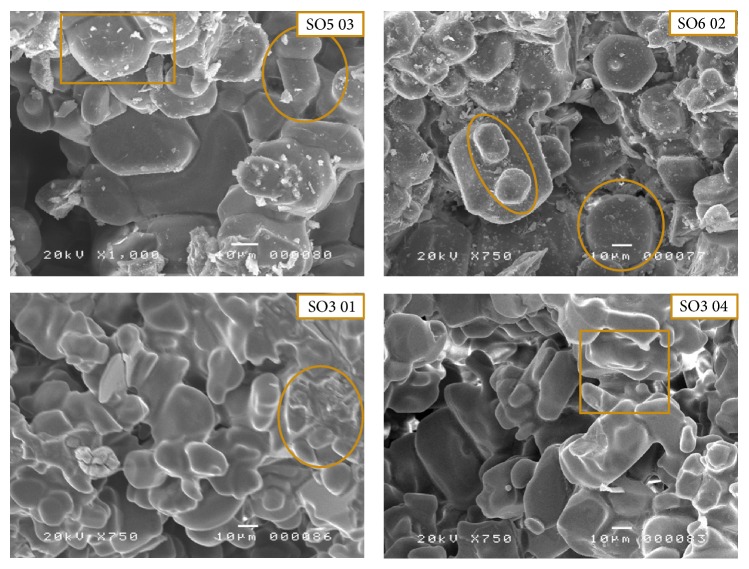
Scanning electron micrograph shows different morphologies of belite and aluminate minerals (C2S and C3A) found in white clinker (belite: SO5 03 and SO6 02; aluminate: SO3 01 and SO3 04).

The minerals are as follows:

- Alite (C3S): the alite crystal is the preferred clinker phase in Portland cement clinker. The formation of alite is very complex. Alite is only formed in the presence of excess CaO, that is, at CaO/SiO_2_ > 1 and is thermodynamically stable above 1250°C [44]. This mineral is found in all the samples in a distinctive crystalline form (Figure 5). The crystallized phases of alite present different morphologies and crystal sizes [43]. However, if the size of the crystals is taken into account, one can easily distinguish the polymorphism of this mineral, (cf. Sample SO4 and SO5) (Figure 5). More precisely, one can observe large, elongated, and well-crystallized crystals which often appear with truncations (SO4 06 and SO4 09 in Figure 5). Image SO5 01 in Figure 5 shows the crystal distribution in the clinker grains. It is a homogeneous distribution where the crystal edges are very clear with an amorphous phase. There were also other well-crystallized crystals (Euhedral alite crystals in clinker void) with smooth sides and clear edges similar to hexagonal shapes (SO5 04 and SO6 04, Figure 5).
- Belite (C2S): another most common and well-known component of clinker is the belite *β*-C2S (monoclinic). In industrial cement production belite is formed in the rotary kiln at 900–1250°C [45]. The basis of belite formation is the reaction between solid CaO and SiO_2_ particles. In our case, this mineral had ovoid or globular shape (SO5 03 and SO6 02, Figure 6). It was not possible to distinguish an individual crystalline structure. The belite formation is closely related to alite formation, that is, if one increases, automatically, the other will be decreased (samples SO2 and SO4. Table 3).
- The tricalcium aluminate (C3A): it was not possible to distinguish well-defined shapes. This mineral appeared as an interstitial phase [46] within the major phases (C3S and C2S) (SO3 01, Figure 6) or vitreous (SO3 04, Figure 6). C3A plays a crucial role in clinkerisation formation, as this phase forms a molten phase at lower temperatures, which influence the development of the chemical and physical properties of the final clinker product greatly.

### 3.2. Defects Associated with the Crystallization

Critical crystallization-associated defects which are visible under the SEM are as follows.

#### 3.2.1. Free Lime

**Figure 7:**
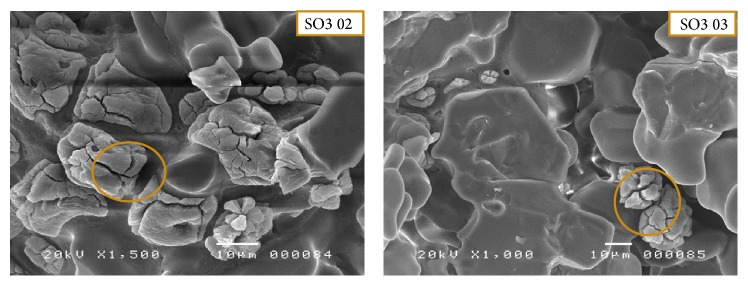
SEM images show the free lime in the grains of white clinker.

**Figure 8:**
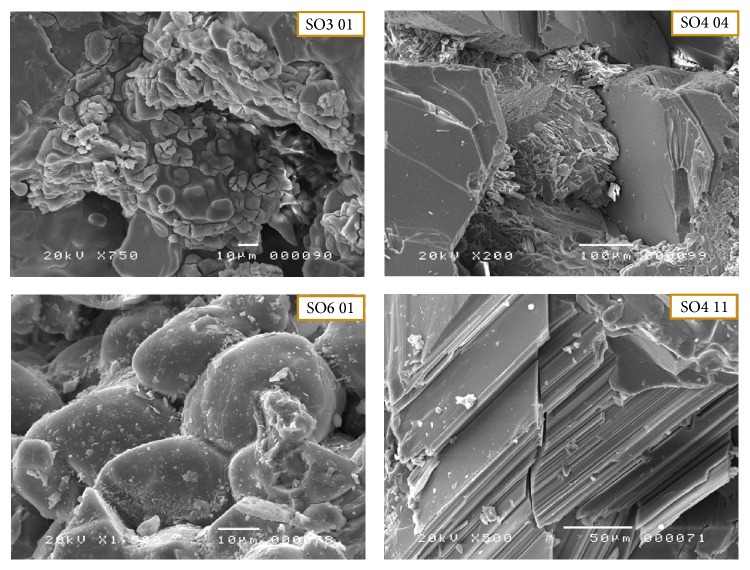
SEM images show some crystallization defects in the mineralogical phases of white cement clinker.

Free lime is an essential component that has a direct influence on the quality of clinker and provides information on the degree of baking. Well-cooked clinkers, resulting from a well measured out raw material and of a sufficiently large size, have less than 2%. Therefore, the C3S concentration, determined by XRD analysis, and the unreacted CaO concentration, determined by XRD and free lime analysis, are followed as a measure of the progress/completeness of alite phase formation. CaO is consumed by the formation of C3S; in fact, the consumption of CaO during the alite formation could result in pores of the size of the original CaO agglomerates, surrounded by a product layer. SEM observations of the SO3 sample showed the presence of a large amount of lime (CaO) which did not react with the silica and alumina (SO3 02, Figure 7 and SO3 01, Figure 8) [15]. This was confirmed by X-ray powder diffraction combined with the Rietveld method. The weight percentage of lime reached 3.8% in the SO3 sample. This proves the poor quality of the clinker studied. This anomaly is due to

(i) high refusal (grinding problem);
(ii) insufficient treatment of raw materials (cold oven);
(iii) dissociation of C3S in C2S due to uncontrolled cooling and the production of CaO (C3S → CaO + C2S);
(iv) gas flow and raw material.

#### 3.2.2. Poor Crystallization

**Figure 9:**
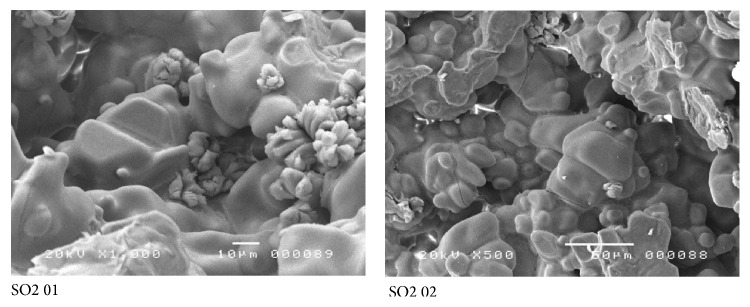
SEM observations show crystals during formation: diagenesis of white clinker crystals.

Poor crystallization is reflected by the presence of incomplete crystalline phases (SO6 01, Figure 8) and gels (SO4 04, Figure 8). The phases are rather rough without well-defined contours. The growth of alite and belite crystals was observed in the melt phase (Figure 9) [46]. This phenomenon is linked primarily to fluctuations in the temperature of the oven (low temperature ≈ 1200–1300°C). This poor crystallization is also evidenced by the appearance of rounded to subrounded shapes with diffuse boundaries between the phases.

#### 3.2.3. The Presence of Lamellar Structures

Leafy and lamellar structures were observed (SO4 11, Figure 8) and accumulated around the alumina silicates or the belite crystals (C2S) which crystallize into lamellar shapes.

## 4. Conclusion

The SEM observations of the poor quality clinker samples (noncompliant with the regulations in force) identified the following points:

- It is possible to distinguish different morphologies (euhedral and anhedral shapes) and sizes of alite within the same sample. This indicates that baking is inhomogeneous in the oven and that the temperature fluctuates at least at the chamber level relative to the formation of alite. It is known from the literature that, at room temperature, only the monoclinic phase exists.
- The presence of free lime, the vitreous phase, and lamellar structures proves the instability of the oven temperature during baking or cooling.
- Excess lime and silica gels also seem to be related to the formulation and even the homogenization of the raw material. In fact, the kaolin is exceptionally rich in silica which results in baking being affected by the uncombined silica gel.
- SEM observation provides information on the degrees of cooking of clinker.

## Figures and Tables

**Table 1:** Average chemical composition of raw materials used for the production of white clinker.

Sample	Al_2_O_3_	CaO	Fe_2_O_3_	K_2_O	MgO	SO_3_	SiO_2_	TiO_2_	LOI	Total
Limestone	0,42	54,97	0,11	0,00	0,04	0,00	1,10	0,015	43,26	99,44
Kaolin	26,07	1,97	0,85	0,15	0,23	0,27	58,52	0,79	10,57	99,65
Sand	2,24	1,72	0,38	0,74	0,16	0,03	91,77	0,25	1,99	99,26

LOI = loss on ignition at 1050°C.

**Table 2:** Geochemical analyses determined by XRF expressed in terms of weight percent of oxides.

Element	Al_2_O_3_	CaO	Fe_2_O_3_	K_2_O	MgO	SO_3_	SiO_2_	TiO_2_	LOI
SO1	3,94	70,20	0,19	0,18	0,25	0,00	23,36	0,10	0,40
SO2	3,66	70,22	0,14	0,18	0,25	0,00	24,28	0,11	0,60
SO3	3,95	70,56	0,17	0,18	0,89	0,10	23,75	0,09	0,33
SO4	3,83	69,85	0,25	0,16	0,25	0,04	24,39	0,13	0,41
SO5	3,95	70,00	0,24	0,17	0,27	0,00	24,40	0,17	0,32
SO6	4,08	70,04	0,31	0,18	0,27	0,00	24,46	0,15	0,53

LOI = loss on ignition at 1050°C.

**Table 3:** Result of Rietveld refinements of the X-ray data. Angular domain: 2*θ* Cu = 10–60.

Phase	Reference	SO1	SO2	SO3	SO4	SO5	SO6
C3S-alite, Nishi et al.	[38]	69,0	85,8	61,6	58,5	70,4	74,3
C2S-beta, belite, (Mumme et al. [39])	[39]	17,8	2,8	22,7	27,6	19,4	16,3
C3A-aluminate-cubic	[9]	7,8	6,4	6,7	7,4	8,3	7,8
Calcium oxide-lime	[40]	1,8	1,4	3,8	0,5	0,2	0,3
Portlandite	[41]	3,2	2,2	3,1	5,8	0,5	0,7
Periclase MgO	[42]	0,4	1,4	2,0	0,2	1,2	0,5
Rwp		15,89	15,00	14,10	14,60	14,90	15,79
Rp		11,69	10,88	10,33	10,84	11,04	11,74
Gof		1,45	1,34	1,27	1,21	1,32	1,49

Rwp, Rp, and Gof: the conventional Rietveld agreement indexes of profile fitting.
